# Socioeconomic Status Is Not Related with Facial Fluctuating Asymmetry: Evidence from Latin-American Populations

**DOI:** 10.1371/journal.pone.0169287

**Published:** 2017-01-06

**Authors:** Mirsha Quinto-Sánchez, Celia Cintas, Caio Cesar Silva de Cerqueira, Virginia Ramallo, Victor Acuña-Alonzo, Kaustubh Adhikari, Lucía Castillo, Jorge Gomez-Valdés, Paola Everardo, Francisco De Avila, Tábita Hünemeier, Claudia Jaramillo, Williams Arias, Macarena Fuentes, Carla Gallo, Giovani Poletti, Lavinia Schuler-Faccini, Maria Cátira Bortolini, Samuel Canizales-Quinteros, Francisco Rothhammer, Gabriel Bedoya, Javier Rosique, Andrés Ruiz-Linares, Rolando González-José

**Affiliations:** 1 Grupo de Investigación en Biología Evolutiva Humana, Instituto Patagónico de Ciencias Sociales y Humanas, Centro Nacional Patagónico, CONICET. Puerto Madryn, Chubut, Argentina; 2 Ciencia Forense, Facultad de Medicina, Universidad Nacional Autónoma de México, Ciudad de México, México; 3 Superintendência da Polícia Técnico-Científica do Estado de São Paulo. Equipe de Perícias Criminalísticas de Ourinhos, São Paulo, Brazil; 4 Department of Genetics, Evolution and Environment, and UCL Genetics Institute, University College London, London, United Kingdom; 5 Escuela Nacional de Antropología e Historia. Instituto Nacional de Antropología e Historia, Ciudad de México, México; 6 Posgrado en Antropología Física, Escuela Nacional de Antropología e Historia, Ciudad de México, México; 7 Departamento de Genética e Biologia Evolutiva, Instituto de Biociências, Universidade de São Paulo; 8 GENMOL, Universidad de Antioquia, Medellín, Colombia; 9 Departamento de Técnología Médica, Facultad de Ciencias de la Salud, Universidad de Tarapacá, Arica, Chile; 10 Laboratorios de Investigación y Desarrollo, Facultad de Ciencias y Filosofía, Universidad Peruana Cayetano Heredia, Lima, Perú; 11 Departamento de Genética, Instituto de Biociências, Universidade Federal do Rio Grande do Sul, Porto Alegre, Brasil; 12 Unidad de Genómica de Poblaciones Aplicada a la Salud, Facultad de Química, UNAM-Instituto Nacional de Medicina Genómica, Ciudad de México, México; 13 Instituto de Alta Investigación Universidad de Tarapacá, Arica, Chile; 14 Departamento de Antropología. Facultad de Ciencias Sociales y Humanas. Universidad de Antioquia, Medellín, Colombia; 15 MOE Key Laboratory of Contemporary Anthropology, Fudan University, Shanghai, China; 16 Aix Marseille Univ, CNRS, EFS, ADES, Marseille, France; Medical University of South Carolina, UNITED STATES

## Abstract

The expression of facial asymmetries has been recurrently related with poverty and/or disadvantaged socioeconomic status. Departing from the developmental instability theory, previous approaches attempted to test the statistical relationship between the stress experienced by individuals grown in poor conditions and an increase in facial and corporal asymmetry. Here we aim to further evaluate such hypothesis on a large sample of admixed Latin Americans individuals by exploring if low socioeconomic status individuals tend to exhibit greater facial fluctuating asymmetry values. To do so, we implement Procrustes analysis of variance and Hierarchical Linear Modelling (HLM) to estimate potential associations between facial fluctuating asymmetry values and socioeconomic status. We report significant relationships between facial fluctuating asymmetry values and age, sex, and genetic ancestry, while socioeconomic status failed to exhibit any strong statistical relationship with facial asymmetry. These results are persistent after the effect of heterozygosity (a proxy for genetic ancestry) is controlled in the model. Our results indicate that, at least on the studied sample, there is no relationship between socioeconomic stress (as intended as low socioeconomic status) and facial asymmetries.

## Introduction

Fluctuating asymmetry (FA) on bilateral human phenotypic attributes has been proposed as a proxy of development instability [1–10]. This idea states that facial asymmetries can be seen as a biomarker of an organism stress during its development, and indirectly accounts for their phenotypic and genetic quality and/or stability [2,11–16], even though some analyses challenge this relationship [17–21]. Based on the pan-adaptationist idea of the “biology of the poverty”, by which humans adjust their surrounding constraints via biologial, social and ideological shifts [22], some publications have tested for variable effects of socioeconomic status (SES) on facial asymmetry features in human populations [7,23,24]. One recent approach, however, reported lower FA scores in the more stressed sub-sample in female European skulls [10]. Moreover, some studies suggested significantly higher FA values in lower SES groups, with males showing higher FA values than females [7] or relatively higher FA values on a sample subjected to higher stress levels [5]. From a diachronic perspective, there is evidence that modern skulls present higher values of FA when compared to medieval ones on a population from Poland [25]. Furthermore, FA has been associated with health status and there is some evidence for higher FA rates in individuals deceased due to degenerative diseases rather than those who suffered infectious diseases, with males exhibiting higher FA values than females [9]. Regarding body and facial measurements, there is a report showing higher values of FA in individuals having lower SES. Foot width, elbow width and knee width appear as the characters displaying greater FA, whereas ear width shows the opposite trend [24]. The same results were obtained in another groups of the same city [26].

Undoubtedly, a large core of publications identified negative consequences related to growing and developing on low SES contexts, and a wide spectrum of outcomes, such as asthma, infections, [27,28] and disease patterns [29] have been described. A recent investigation on a large European sample failed to detect any relationship among childhood health status and FA [30]. However, and considering that the development and genetic basis of FA is largely unknown [31] and taking into account that craniofacial traits are determined by a complex genotype-phenotype map [32–38], the using of FA as an indicator of developmental stress due to SES context deserves further research.

In this context, we postulate that before establishing hypothesis based on the assumption that fluctuating asymmetry is a proxy of developmental instability, some important factors deserve attention. For instance, it should be noted that some degree of directional or fluctuating facial asymmetry is normal (e.g. non-pathological) at the population level, even among healthy individuals see [39–41]. Also, the usefulness of FA as an indicator of individual developmental stability is poor unless the variance of developmental stability is extremely large [42]. In addition, large samples are usually needed to detect stress effects on FA and measurements need to be made at least twice in the order to test for reliability of the asymmetry parameter [43,44]. Finally, even if the relationships between FA, stress and developmental stability are poorly understood, it can confidently be said that FA is not a general, and sensitive, indicator of stress.

Considering all the above, the question arises if there is some detectable effect of SES inequality on the patterns of FA. In this context, the establishment of human facial features or expressions as biological adaptations requires a rigorous review of current knowledge on the “normal” variation patterns, which is a basic preliminary step to test any evolutionary hypothesis [45].

### Latin America as “natural experiment” to test asymmetries versus socio-economic status hypotheses

Historically, the region has been marked by continuous, differential and diverse intra and intercontinental migration events [46]. Such migratory history is accompanied by unequal rates of socioeconomic development [47], sustained in the pervasive inequality inherent to Latin America [48]. Even when most Latin-American countries enhanced the socioeconomic status of their populations during the last decade, still there is an annual reduction of 7 percent points of land covered by forest, a low and increasing unemployment ratio, an 80% of their population live in cities [49], and 167 millions (28%) of population is still under poverty and 66 millions under extreme poverty conditions. Additionally, Latin America presents higher prevalence of infectious and metabolic diseases [50]. Overall, and in contrast to studies made on other regions and contexts [5,7,9,10,24,25], the abovementioned socioeconomic landscape provides a proper scenario to explore the central tendency and variation of FA across a wide range of socioeconomic levels established through wide geographic ranges.

In this paper, we aim to test if, besides the potential effects of genetic admixture on it, FFA scores are linked to multivariate SES inequality on a sample of urban individuals belonging to five admixed Latin American populations. Note that, instead of developing a classic approach aimed to test if extreme poor conditions trigger some degree of FA, we focus on the “normal” observed range of SES variations on modern, urban Latin-American populations.

## Subjects and Methods

### The sample

As part of the CANDELA initiative [51,52], we recruited 2,019 volunteers between 18 and 63 years (mean = 25.73 sd = 6.64; females = 850 mean = 25.05 sd = 6.23; males = 1165 mean = 26.22 sd = 6.88), from ten Latin-American cities: Mexico City (Mexico), Medellin (Colombia), Lima (Peru), Arica (Chile) and Porto Alegre, Jequié, Porto Velho, Sao Gabriel, Cândido Godoi and Imbé (all in Brazil). The inclusion-exclusion criteria were age range (at least 18 years old), lack of antecedents of craniofacial dismorphologies, orthodontics treatments or severe facial trauma. Further sample details are provided in Table 1 and the extended database is provided as S2 Table. Approvals provided by Ethics Committees (Universidad Nacional Auntónoma de México, Escuela Nacional de Antropología e Historia, Universidade Federal do Rio Grande do Sul, Universidad de Chile, Universidad Peruana Cayetano Heredia and Universidad de Antioquia) were obtained prior the data collection, and an informed consent were signed for each participant before genetic, socioeconomic and facial phenotype data was collected [51,52].

**Table 1:** Sample details concerning age, gender and country for a total of 2,018 volunteers.

	Age					
	Young adult (18–20)		Early adult (20–40)		Middle adult (40–60)	
	Sex					
Country	f	m	f	m	f	m
Brazil	28	13	129	65	5	7
Chile	3	115	104	525	6	40
Colombia	106	60	208	173	0	0
Mexico	93	40	144	111	0	0
Peru	1	3	24	15	0	0
Totals	231	231	609	889	11	47

All covariates were taken as continuous variables (sex was assessed taking the discriminant function axis between sexes). For tabulation of the Table 1, age was discretized following Sigelman and Rider [53] as young adult (18–20 years old), early adult (20–40 years old) and middle adult (40–60 years old).

### Facial 3D phenotyping

**Fig 1:**
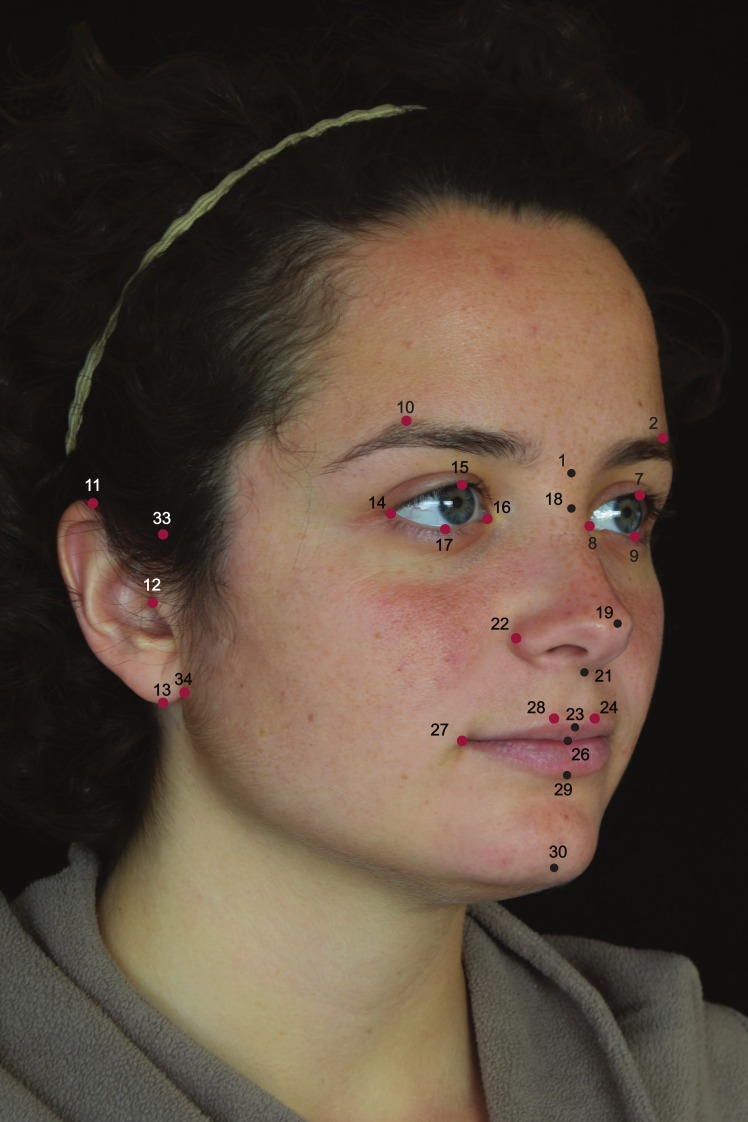
Anatomical location of the 34 landmarks used in this study depicted on a fronto-lateral view of the face (see Table 2 for definitions). According to the ethic approval and written informed consent of CANDELA Project, pictures of volunteers cannot be provided in scientific publications or websites. The image presented here belongs to a collaborator who has given written informed consent (as outlined in PLOS consent form) to publish this figure, and was taken following the standard CANDELA protocols.

Facial shape was recorded following scientific photographic protocols described in detail in references [51] and [52]. Upon these images, two observers (MQS and LC) placed a set of 34 standard facial landmarks (Fig 1, Table 2) using the Photomodeler software (http://www.photomodeler.com/ Eos Systems Inc, Vancouver, Canada). As described elsewhere [52], this platform corrects for any lens distortion automatically, and we have followed the standard recommendations for quality and accuracy provided by the software. Scale factor was assessed using the nasion-gnathion distance measured directly on the individuals using a standard anthropometric caliper.

**Table 2:** Facial landmark anatomical definitions (see Fig 1 for anatomic-spatial reference).

No.	Name	Definition
Sagitals		
1	Glabella	The smooth area between the eyebrows just above the nose
18	Nasion (sellion)	The midpoint of the nasofrontal suture
19	Pronasal	The most protruded point of the nasal tip
20	Subnasal	The junction between the lower border of the nasal septum and the cutaneous portion of the upper lip in the midline
23	Labiale superious	The midpoint of the vermilion border of the upper lip
26	Stomion	The midpoint of the labial fissure when the lips are closed naturally
29	Labiale inferious	The midpoint of the vermillion border of the lower lip
30	Gnathion	The lowest point in the midline on the lower border of the chin
Bilaterals		
2,10	Frontotemporale	The most medial point on the temporal crest of the frontal bone
3,11	Superaurale	The highest point of the free margin of the ear
4,12	Tragion	The tip of tragus
5,13	Subaurale	The lowest point of the ear lobe
6,16	Exocanthion	The outer corner of the eye fissure where the eyelids meet
7,15	Palpebrale superiorus	The superior point of the eyelid
8, 14	Endocanthion	The inner corner of the eye fissure where the eyelids meet
9, 17	Palpebrale inferiorus	The inferior point of the eyelid
21,22	Alare	The most lateral point on the nasal alar
24,28	crista philtre (upper lip point)	Highest point of the upper vermillion
25,27	Cheilion	The outer corner of the mouth where the outer edges of the upper and lower vermilions meet
31,33	Otobasion superiorious	The superior point on the union of the lobule and the head
32,34	Otobasion inferiorous	The basal point on the union of the lobule and the head

### Genomic data

**Fig 2:**
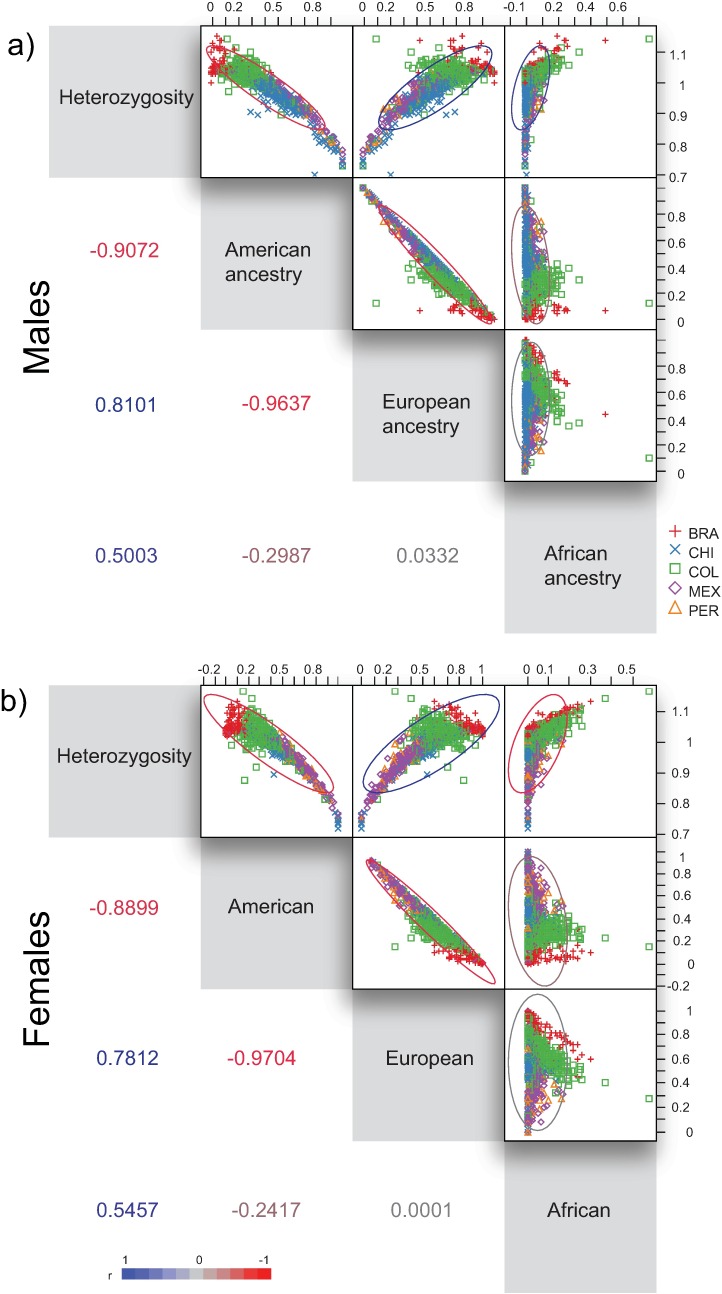
Matrix depicting correlations among wide-genome heterozygosity scores and genetic ancestry estimations. a) Males, b) Females. Ellipses account for the 95% of the variance and the color depicts the r-value magnitude (see right-bottom scale for reference). The corresponding r-values are placed in the lower triangle of the matrix.

From a blood sample taken from each volunteer we obtained genomic data involving 730,525 SNPs (single nucleotide polymorphisms) [51,52]. After quality control of the genetic data and the exclusion of markers showing linkage disequilibrium, we used 90,000 SNPs in order to perform ancestry estimations and obtain genome-wide average heterozygosity values using PLINK [54,55]. Heterozygosity is calculated as 1 –homozygosity [52]. We correlated heterozygosity against genetic ancestry estimations in order to characterize the distribution of this variable in our sample (Table 3, Fig 2). The correlations among parental genetic ancestries and heterozygosity provided highly significant pairwise r-values (p <0.0001), thus bringing support to using heterozygosity as an indicator of genetic ancestry.

**Table 3:** Pairwise correlations and corresponding p-values for heterozygosity against the parental genetic ancestry values.

Variable		Correlation	Lower 95%	Upper 95%	p-value
American	Heterozygosity	-0.898	-0.9061	-0.8892	< .0001
European	Heterozygosity	0.7938	0.7771	0.8094	< .0001
European	American	-0.9668	-0.9695	-0.9638	< .0001
African	Heterozygosity	0.5298	0.4977	0.5604	< .0001
African	American	-0.2789	-0.3186	-0.2382	< .0001
African	European	0.0242	-0.0194	0.0677	0.2758

### Repeatability and error measurement tests

Due to its statistical properties, fluctuating asymmetry can be easily confounded with measurement errors [56]. Thus, an a priori estimation of the magnitude and pattern of error is mandatory in order to guarantee that deviations from symmetry are due to biological factors, rather than to methodological artifacts. Inter and intra observer error and repeatability assessments were performed on the basis of replicated data. To measure inter-observer error both observers generated multiple digitizing rounds on a single male individual, during four separate sessions distributed across three months. After the Procrustes superimposition, the Euclidean distance of each landmark to its respective centroid was computed and, for each observer, landmark deviations were calculated relative to the observer landmark mean. Following Singleton [57], mean deviations were calculated for individual landmarks and subsequently averaged to give a mean deviation for each observer across all landmarks. One-way analysis of variance was performed for each landmark by observer and the root mean squares were evaluated. The root of the within-groups mean squares (root mean square error) is an estimation of the intraobserver error [58], while the root of between-groups mean squares denotes interobserver error.

Data repeatability was estimated on the basis of replicated data belonging to 200 individuals taken from the five countries and both sexes. The mean square values resulting from a Procrustes ANOVA performed on this data subset can be considered as a variance score that can be compared to the FA and DA variances. If the used protocol guarantees data repeatibillity, then the magnitude of the error term would be considerably lower than the individual, DA and FA effects [56].

### Estimation of the facial fluctuating asymmetry scores

We characterized the asymmetric component of shape variation in the facial phenotype using Procrustes ANOVA and MANOVA designs [56], following methods used on a recent paper [52]. Facial landmark configurations with object symmetry can be dissected into components of symmetric and asymmetric variation by Procrustes superimposition of the original configurations and their mirror images [59]. MorphoJ [60] provides individual facial fluctuating asymmetry (FFA) scores, that is a metric aimed to quantify how the individual fluctuates around its own mean asymmetry see more details in ref. [59] and [61]. The Procrustes ANOVA model estimates the significance of an individual, side, individual-by-side interaction and measurement error effects, accounting for individual, directional asymmetry (DA), FA and error variation, respectively [56,61]. P-values were calculated using a permutation test based on 100,000 iterations of the original data.

### Statistical analyses related to test association of SES and facial asymmetry

Departing from the intrinsic complexity and multifactorial, non linear nature of the SES [62] we approached a model on the basis of three variables available in the CANDELA survey: fixed monthly salary (FMS), frequency of domestic appliances by home, and education (schooling). We use a multivariate composite measure of SES using a Principal Component Analysis approach, following refs. [62] and [63], in order to obtain a wealth index (WI) with all PC scores exhibiting eigenvalues higher than unit. Then we use these PC scores as a proxy for SES variation in the sample. FMS was measured in the domestic currency of each country and then converted to US dollars in order to homogenize the variable. The range of this variable goes from 8.99 to 6.149 dollars per month (mean = 805.65, sd = 678.23). The CANDELA survey registered the frequency of all domestic appliances, including 14 elements such as cars, radio, TV, bicycles, etc. Finally, education was assessed as a meristic variable containing four categories: primary, secondary, universitary (or graduate) and postgraduate. Whenever possible, we preferred to maintain the continuous nature of the SES variables (statistical support of the SES indexes is presented in S1 Text).

The relationship between FFA scores and SES was explored using a Hierarchical Linear Model (HLM) implemented in SPSS v. 12. Thus, individuals were “nested” within sub-populations (i.e. Brazil, Chile, Colombia, Mexico, Peru) in order to circumvent any among-country effect on the SES variables, and to evaluate significant effects of the FFA scores by country and sex. Age, sex, BMI, melanin index, heterozygosity, genetic ancestry and SES variables where considered as main effects in the model. On a previous paper on the same populations [52] we reported that genome heterozygosity is negatively correlated with FFA scores and, in consequence, is highly related with genetic ancestry. Thus, to further refine our analysis on the potential relationship among FFA and SES, we replicated all the abovementioned analyses on the residuals of the regression of FFA scores on the heterozygosity values.

## Results

### Repeatability and error measurement tests

The ANOVA approach compute on the distances to the centroid proposed by [57] provides the root mean square errors, which can be intended as intra-observer error, [58], whereas the root of the mean squares among groups explains inter-observeer error. Our error tests results are presented in Table 4, and they show that, to the exception of the first session (December), the error percentage (%RMSE) is greater for the intra-observer than for the inter observer error. Specifically, it can be noted that the inter-observer error diminishes across the consecutive sessions (from 1.4884 to 0.9172), indicating a better performance due to the increased training of both observers. The mean value for the inter-observer distance is 0.0373, which is lower than any of the inter-individual distances for any observer (Table 5). Also, the maximum inter-observer distance (0.0739) is well below the average intra-observer error for both observers.

**Table 4:** Inter- and intraobserver mean errors across three digitizing sessions (February, January and December). RMSE = Root of the mean square error (see text for details).

February	RMSE			%RMSE		
	min	max	mean	min	Max	mean
Interobserver	0	0.0053	0.0009	0	1.6995	0.9172
Intraobserver	0	0.0032	0.0007	0.3005	2	1.0828
January	RMSE			%RMSE		
	min	max	mean	min	Max	mean
Interobserver	0	0.0033	0.0007	0.1713	1.411	0.908
Intraobserver	0	0.0026	0.0007	0.589	1.8287	1.092
December	RMSE			%RMSE		
	min	max	mean	min	Max	mean
Interobserver	0	0.0149	0.0043	0.2665	1.8766	1.4884
Intraobserver	0	0.0028	0.0009	0.1234	1.7335	0.5116

**Table 5:** Central tendency and dispersal statistics for within and between-observer differences.

effect	min	max	sd	mean	median	mode
Observer 1	0.0410	0.1719	0.0170	0.0827	0.0806	0.0575
Observer 2	0.0490	0.1505	0.0171	0.0794	0.0765	0.0833
O1-O2	0.0257	0.0739	0.0169	0.0373	0.0316	0.0833
Totals	0.0257	0.1719	0.0169	0.0828	0.0808	0.0833

The Procrustes ANOVA repeatability essay, performed on the 200 replicates, indicated that the error term provided the lowest mean square values in relation to variation due to sex, individual, DA, and FA (Table 6). Note that the error term is slightly smaller than FA, a result that both, guarantee the reliability of our analyses, and reinforces our general claim for caution when using FA as a proxy to developmental instability (see below). This cautionary note is particularly important when FA is obtained from small sample sizes and error levels are not evaluated. The common sense indicates that the error should be smaller than FA, but there is no rule-of-thumb stating how smaller the error needs to be. Given the large sample size used here and the associated metadata (genomics, phenotypes, SES variables, etc.) that we have compiled to perform this study, we have no ways of comparing our error levels with similar analyses. However, what is clear is that error levels are not above the magnitude of FA in our sample.

**Table 6:** Repeatability essay: Procrustes ANOVA results with sex as covariate.

Procrustes ANOVA					
Effect	SS	MS	df	F	P (param.)
Sex	0.4987866	0.0097801	51	163.98	< .0001
Individual	12.6538792	0.0000596	212160	6.64	< .0001
DA	0.0746448	0.0016965	44	188.74	< .0001
FA	1.6456741	0.0000090	183084	1.24	< .0001
Error	0.1132016	0.0000072	15675		

Considering the intra/inter-observer error and repeatability patterns described above, and the relatively large size of the faces studied here, these margins of error and repeatability were considered acceptable.

### SES and facial asymmetry

The Hierarchical Linear Model (HLM) performed on both, heterozygosity corrected and uncorrected individual FFA scores provided a significant dependence of FFA scores on age, sex, BMI, melanin index and heterozygosity (for uncorrected data). Specifically, both corrected and uncorrected FFA increases with age in both sexes. Additionally, independently of the heterozygosity effect, FFA scores increases with BMI in both sexes, showing a marked effect in males (see S1 Table).

We have detected a moderated association with PC3 (p = 0.028) and PC4 (p = 0.036) of the WI (Table 7). The PC1_WI_ resumes 24.44% of the total variance in the sample, and the positive values of this PC are characterized by the possession of TV, video, bathroom, car and radio. PC2_WI_ explains a 9.97% of the total variance and is related to FMS, education and possession of freezer. The two significant principal components, in terms of its association to FFA in the HLM model explain collectively a 14% of the total variation. Specifically, PC3_WI_ explains a 7.72% of the total variance and is mainly explained by fridge and education in the positive values versus having a vacuum and bike in the negative scores. Finally, PC4 _WI_ accounts for 6.30% of the total variance and is related to having a dishwasher in the positive axis and a motorcycle for the negative quadrant (see S1 Text for details in the loading matrix of PCA_WI_). Considering both, the small variance explained by PC3_WI_ and PC4_WI_ added to the fact that these PCs do not seem to discriminate among high and low wealth status, but on the pattern of acquisition or possession of specific goods (e.g. some items are sorted in the positive and others in the negative values of these PCs), we cannot state that there is a relationship among increased levels of asymmetry and low socioeconomic status (Table 7). This general pattern of results is not altered after correction of FFA values for heterozygosity.

**Table 7:** Results of HLM for FFA scores against age, sex, BMI, genetic ancestry and PC scores for wealth index. For simplicity, and considering the high correlation between both data sets, results are presented only for heterozygosity-corrected data (blue-colored cells indicate significant effects at p< = 0.01).

Heterozygosity corrected FFA scores					
	Estimation	sd	df	t	p
Age	0.022	0.002	5,227.956	8.781	0.000
Sex	-0.125	0.030	5,222.607	-4.214	0.000
BMI	-0.015	0.004	5,227.425	-0.387	0.000
Melanine	-0.011	0.003	5,226.054	-3.588	0.000
European	0.196	0.104	3,590.690	1.882	0.060
African	1.387	0.305	5,152.044	4.548	0.000
PC1 WI	0.028	0.014	5,147.350	1.949	0.051
PC2 WI	-0.026	0.016	5,213.821	-1.648	0.100
PC3 WI	0.038	0.017	5,138.595	2.195	0.028
PC4 WI	-0.029	0.014	5,212.806	-2.094	0.036

**Fig 3:**
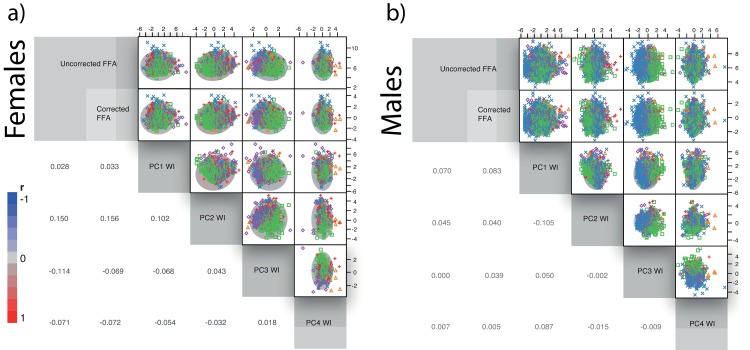
Matrix of correlation of FFA scores (corrected and uncorrected for heterozygosity effects) against SES variables (PCA-WI) by sex. a) Females b) Males. Raw and corrected FFA scores are presented in the left and right columns respectively. Ellipses account for the 95% of the variance and the color depicts the r-value magnitude (see left-bottom scale for reference). Lower triangle of the matrix indicates the r-value correlation scores.

Matrix graphs displaying the pairwise comparisons of FFA values and SES shows non-association among these variables and even a pattern of decreasing FFA as SES values increase (Fig 3). Some of these correlations were significant but the explained variance is very small.

**Fig 4:**
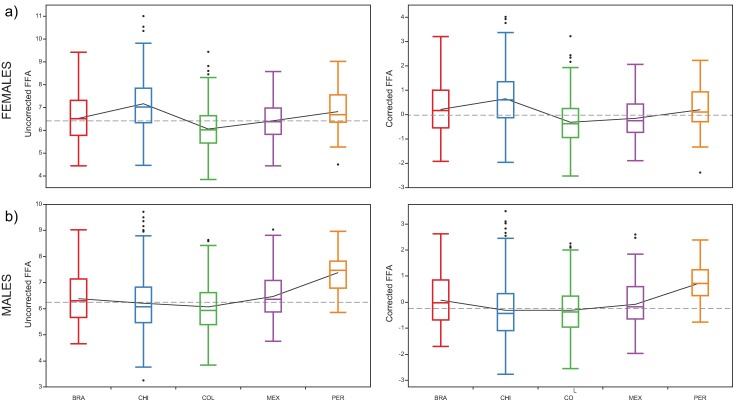
Boxplots of distribution of the FFA scores by country and sex. The dotted line represents the grand mean, the continuous black line connect means among countries. a) Females, b) males. Black points represent outlier values. The left column plots represent raw FFA scores and the right ones the FFA scores corrected for the heterozygosity effects.

Regarding among-country variations, Peru showed the higher FFA values of the sample and Colombia the lower ones in males, whereas the highest FFA values in females were found in Chile (Fig 4, see S1 Table for details on distributions of FFA scores by sex and country). Finally, univariate correlations of FFA scores and PC scores of WI were significant in Females (PC2 and PC3) and males (PC1), but note that these PCs explain less than 10% of the variance.

## Discussion

This study is focused on exploring the potential association among facial asymmetries and SES indicators on a large sample of Latin American admixed samples collected on different Latin-American countries. Following previous recommendations [56,64], the effects of within and between observer error measurement were quantified, in order to guarantee that these effects are negligible in relation to asymmetry values, which is the case in our sample.

Our results indicated significant association of FFA scores with specific covariates such as age, BMI, country and heterozygosity. As previous approaches [52], we identified a strong association of patterns of asymmetry with American and European ancestry, as well as a negative correlation among FFA values and heterozygosity.

Increasing FFA with age is in concordance with previous reports showing ontogenetic increase of skeletal fluctuating asymmetry in Rhesus macaques and humans [65–67]. FFA is also dependent on sex in our sample, being higher in females. Previous estimations of dependence of facial asymmetries on sex are quite contradictory, with some papers reporting statistical differences [10,68–70], and others reporting null or weak sex-effects [6,40,71]. Additionally, some analyses suggest higher FFA values on males [7,66,72], while others are concordant with our results, showing stronger asymmetries in females [10,40].

### FFAs and SES variables

Facial asymmetry was correlated with PC3 and PC4 of a principal component analysis aimed to synthesize a wealth index (Table 4), but these correlations are low in general and do not seem to support any association between asymmetry and socio-economic status since low PCs in the wealth index approach seem to reflect a pattern of good consumption rather the magnitude of it. Because of the complexity of the phenotype under study, an important remark regarding these results is the general sample size achieved. Note that all previous surveys aimed to test for associations among asymmetry and SES are based on samples sizes of 80 to 392 individuals [9,10,23,25], while our sample exceeds by 6 or 15 times these values.

Our core results contradict the previous statement that lower SES is associated to higher facial asymmetries [5,7,23,25,73]. A possible explanation to this discordance is that our sample does not cover cases of extreme poverty (below US$ 8,00 per month), but note that previous reports do not provide specific details on economic income. Conversely, our sample includes detailed indicators of SES and indeed covers a wide range of SES on a large and supervised sample. Specifically, we detected no association between FFA and SES, and individuals with low SES scores do not statistically differ in terms of FFA from those with higher SES. This suggests that high asymmetry values are not characteristic or intrinsic to groups with less income and the allegedly concomitant instability they could suffer during its development.

Some previous reports suggested a relationship among asymmetric facial shape and lower incomes. For instance, Özener [73], analyzing seven body measurements stated that “according to the results, FA is higher in individuals who have lower socioeconomic status, and who, in turn, are subject to environmental stresses”. Foot, elbow and knee appear as the more asymmetric characters [73]. Other reports found a significant relationship of female skull fluctuating asymmetries with lesser SES structure on a more stressed population [10]. Additionally, some authors reported significantly higher FA values in lower SES groups, with males showing higher values than females [7]. Theoretically, it is assumed that impaired SES status is associated to worse life conditions and the consequent developmental instability it would confer. However, a straightforward and empirically based relationship among SES and health conditions is still to be proved. For instance, a recent study in Costa Rican individuals displaying better education and FMS indicators showed poorer health indicators such mortality and metabolic syndrome [74]. In contrast, indexes related to life quality such as functional or cognitive incapacities, physic fragility and depression appeared to be exacerbated in lower SES sub-samples [74]. Factors of cardiovascular risk, diabetes and cholesterol are not related to SES, but hypertension and obesity are more frequent in individuals with higher SES index [74]. Smoking or low exercise-levels are frequent on low SES sub-samples, but highly caloric diets are more ubiquitous among high SES individuals [74]. This complex pattern is indicative of the intrinsic complexity of measuring SES and its impacts on health and phenotypic conditions, including developmental stability/instability. This difficulty increases if large sample sizes are absent, or important factors underlying asymmetries, such as genetic ancestry, are not taken into account. To sum up, individuals occupying both extremes of the socioeconomic variation display great variation on the pattern of the abovementioned “biomedical” phenotypes, indicating that caution is needed when stating hypothesis linking low SES, developmental instability, and FFA and other phenotype as an univocal phenomenon. Such conceptual link is strongly based on the social determinants of health [75], which traditionally associates better health position to social status, an idea that was extrapolated to the relationship among SES and asymmetry. Currently, biological and social scientists agree about the consequences of poverty and social inequality, especially during the peri-natal period and early childhood [76] and regarding low birthweight (defined as less than 2,500 grams). These seem to be clear examples of the biological impact of social inequality that can trigger serious biomedical effects. What our results specifically question is the using of facial asymmetries as an indicator of a developmental instability due to low of higher SES. In other words, we argue that the main statements of the “biology of the poverty” [22], cannot be applied to facial characters, as can be for other characters such as diseases or infections [27–29,50,77].

The CANDELA sample provides an interesting context to test such hypothesis, since pervasive inequality inherent to Latin America [48], marked by continuous, differential, diverse international migration and combined unequal development [46,47] enables comparisons of a wide range of SES scores and their putative associations with asymmetries. In addition, our multidimensional approach to SES guarantees a more accurate and precise indicator of individual and population SES status, as previously stated [78]. Finally, our metadata sample enables the simultaneous exploration and consequent statistical control of heterozygosity as an alternative and complementary source of FFA variation. On a recent paper we have obtained a negative correlation among heterozygosity and facial asymmetries, suggesting that admixed populations may exhibit a greater response to the putative developmental stress implicit in the admixture event among three populations [79]. Our results on the relationship between SES and FFA are robust in the sense that the lack of statistical association remains after the effects of genomic ancestry are removed from data.

## Conclusion

Our approach based on the comparison among multidimensional socioeconomic wealth status indicators and facial fluctuating asymmetries indicates that there is no relationship between both variables. In other words, belonging to the most vulnerable socioeconomic groups is not related to displaying greater asymmetries. The range of asymmetry variance observed in the higher SES subsample may suggest that the responses to developmental stability/instability is not determined in a mechanistic way; rather, it would be driven by many non lineal factors.

## Supporting Information

S1 Table Sample characteristics regarding sample sizes, FFA mean values, standard deviation and variance by country and sex for heterozygosity corrected and uncorrected data. (DOCX)

S2 Table Database: raw data excel file. (XLSX)

S1 Text Computation of a Wealth Index multivariate estimator. (DOCX)

## References

[1] Livshits G, Kobyliansky E. Fluctuating asymmetry as a possible measure of developmental homeostasis in humans: a review. Hum Biol. 1991;63(4):441–66. 1889795

[2] Scheib JE, Gangestad SW, Thornhill R. Facial attractiveness, symmetry and cues of good genes. Proc Biol Sci. 1999;266(1431):1913–7. 10.1098/rspb.1999.0866 10535106 PMC1690211

[3] Milne BJ, Belsky J, Poulton R, Thomson WM, Caspi A, Kieser J. Fluctuating asymmetry and physical health among young adults. Evol Hum Behav. 2003;24(1):53–63.

[4] Simmons LW. Are human preferences for facial symmetry focused on signals of developmental instability? Behav Ecol. 2004;15(5):864–71.

[5] DeLeon VB. Fluctuating asymmetry and stress in a medieval Nubian population. Am J Phys Anthropol. 2007;132(4):520–34. 10.1002/ajpa.20549 17243154

[6] Little AC, Jones BC, Waitt C, Tiddeman BP, Feinberg DR, Perrett DI, et al Symmetry is related to sexual dimorphism in faces: data across culture and species. PLoS One. 2008; 7;3(5):e2106 10.1371/journal.pone.0002106 18461131 PMC2329856

[7] Özener B. Brief communication: Facial fluctuating asymmetry as a marker of sex differences of the response to phenotypic stresses. Am J Phys Anthropol. 2010;143(2):321–4. 10.1002/ajpa.21357 20623680

[8] Özener B, Fink B. Facial symmetry in young girls and boys from a slum and a control area of Ankara, Turkey. Evol Hum Behav. Elsevier; 2010;31(6):436–41.

[9] Weisensee KE. Assessing the relationship between fluctuating asymmetry and cause of death in skeletal remains: a test of the developmental origins of health and disease hypothesis. Am J Hum Biol. 2013;25(3):411–7. 10.1002/ajhb.22390 23559481

[10] Bigoni L, Krajíček V, Sládek V, Velemínský P, Velemínská J. Skull shape asymmetry and the socioeconomic structure of an early medieval central European society. Am J Phys Anthropol. 2013;150(3):349–64. 10.1002/ajpa.22210 23283725

[11] Parsons PA. Fluctuating asymmetry: a biological monitor of environmental and genomic stress. Heredity (Edinb). 1992;68(4):361–4. 1563968 10.1038/hdy.1992.51

[12] Møller A, Thornhill R. Developmental stability is heritable. J Evol Biol. 1997;10:69–76.

[13] Rhodes G, Zebrowitz LA, Clark A, Kalick SM, Hightower A, McKay R. Do facial averageness and symmetry signal health? Evol Hum Behav. 2001;22(1):31–46. 11182573 10.1016/s1090-5138(00)00060-x

[14] Rhodes G, Proffitt F, Grady JM, Sumich A. Facial symmetry and the perception of beauty. Psychon Bull Rev. 1998;5(4):659–69.

[15] Perrett DI, Burt DM, Penton-Voak IS, Lee KJ, Rowland DA, Edwards R. Symmetry and Human Facial Attractiveness. Evol Hum Behav. 1999;20(5):295–307.

[16] Zaidel DW, Hessamian M. Asymmetry and Symmetry in the Beauty of Human Faces. Symmetry (Basel). Molecular Diversity Preservation International; 2010;2(1):136–49.

[17] McKenzie JA, Clarke GM. Diazinon resistance, fluctuating asymmetry and fitness in the Australian sheep blowfly, lucilia cuprina. Genetics. 1988;120(1):213–20. 17246476 10.1093/genetics/120.1.213 PMC1203491

[18] Graham JH, Freeman DC, Emlen JM. Antisymmetry, directional asymmetry, and dynamic morphogenesis. Genetica. 1993;89(1–3):121–37.

[19] Björklund M, Merilä J. Why some measures of fluctuating asymmetry are so sensitive to measurement error. Ann Zool Fennici. 1997;34:133–7.

[20] Lens L, Van Dongen S, Kark S, Matthysen E. Fluctuating asymmetry as an indicator of fitness: can we bridge the gap between studies? Biol Rev Camb Philos Soc. 2002;77(1):27–38. 11911372 10.1017/s1464793101005796

[21] Bjorksten T, Fowler K, Pomiankowski A. What does sexual trait FA tell us about stress? Tree. 2000;15(4):163–6. 10717689 10.1016/s0169-5347(99)01788-7

[22] Tomas R. The evolution of human adaptability paradigms: towards a biology of poverty In: Goodman A, Leatherman T, editors. Building a New Biocultural Synthesis: Political-economic Perspectives on Human Biology. Michigan: The University of Michigan Press; 1998 p. 43–73.

[23] Özener B. Fluctuating and directional asymmetry in young human males: effect of heavy working condition and socioeconomic status. Am J Phys Anthropol. 2010;143(1):112–20. 10.1002/ajpa.21300 20734438

[24] Özener B. Does urban poverty increase body fluctuating asymmetry? Coll Antropol. 2011;35(4):1001–5. 22397230

[25] Gawlikowska A, Szczurowski J, Czerwiński F, Miklaszewska D, Adamiec E, Dzieciołowska E. The fluctuating asymmetry of medieval and modern human skulls. Homo. 2007;58(2):159–72. 10.1016/j.jchb.2006.10.001 17445814

[26] Özener B. Tall men with medium body fat mass percentage display more developmental stability. Homo. 2010;61(6):459–66. 10.1016/j.jchb.2010.09.004 20970797

[27] Chen E, Martin AD, Matthews KA. Socioeconomic status and health: do gradients differ within childhood and adolescence? Soc Sci Med. 2006;62(9):2161–70. 10.1016/j.socscimed.2005.08.054 16213644

[28] Evans GW, Kim P. Childhood poverty and health: cumulative risk exposure and stress dysregulation. Psychol Sci. 2007;18(11):953–7. 10.1111/j.1467-9280.2007.02008.x 17958708

[29] Shonkoff JP, Boyce WT, McEwen BS. Neuroscience, molecular biology, and the childhood roots of health disparities: building a new framework for health promotion and disease prevention. JAMA. American Medical Association; 2009;301(21):2252–9. 10.1001/jama.2009.754 19491187

[30] Pound N, Lawson DW, Toma AM, Richmond S, Zhurov AI, Penton-Voak IS. Facial fluctuating asymmetry is not associated with childhood ill-health in a large British cohort study. Proc R Soc Lond B Biol Sci. 20014;281:e20141639. 10.1098/rspb.2014.1639 PMC4150332 25122232

[31] Klingenberg CP, Nijhout HF. Genetics of fluctuating asymmetry: A developmental model ofdevelopmental instability. Evolution (N Y). 1999;53(2):358–75. 10.1111/j.1558-5646.1999.tb03772.x 28565420

[32] Liu F, van der Lijn F, Schurmann C, Zhu G, Chakravarty MM, Hysi PG, et al A genome-wide association study identifies five loci influencing facial morphology in Europeans. PLoS Genet. 2012;8(9):e1002932 10.1371/journal.pgen.1002932 23028347 PMC3441666

[33] Paternoster L, Zhurov AI, Toma AM, Kemp JP, St Pourcain B, Timpson NJ, et al Genome-wide association study of three-dimensional facial morphology identifies a variant in PAX3 associated with nasion position. Am J Hum Genet. The American Society of Human Genetics; 2012;90(3):478–85. 10.1016/j.ajhg.2011.12.021 22341974 PMC3309180

[34] Peng S, Tan J, Hu S, Zhou H, Guo J, Jin L, et al Detecting Genetic Association of Common Human Facial Morphological Variation Using High Density 3D Image Registration. PLoS Comput Biol. 2013;9(12):e1003375 10.1371/journal.pcbi.1003375 24339768 PMC3854494

[35] Mezey JG, Cheverud JM, Wagner GP. Is the Genotype-Phenotype Map Modular?: A Statistical Approach Using Mouse Quantitative Trait Loci Data. Genetics. 2000;156(1):305–11. 10978294 10.1093/genetics/156.1.305 PMC1461239

[36] Adhikari K, Reales G, Smith AJP, Konka E, Palmen J, Quinto-Sanchez M, et al A genome-wide association study identifies multiple loci for variation in human ear morphology. Nat Commun. Nature Publishing Group; 2015;6:7500 10.1038/ncomms8500 26105758 PMC4491814

[37] Adhikari K, Fontanil T, Cal S, Mendoza-Revilla J, Fuentes-Guajardo M, Chacón-Duque J-C, et al A genome-wide association scan in admixed Latin Americans identifies loci influencing facial and scalp hair features. Nat Commun. 2016;7:10815 10.1038/ncomms10815 26926045 PMC4773514

[38] Adhikari K, Fuentes-Guajardo M, Quinto-Sánchez M, Mendoza-Revilla J, Camilo Chacón-Duque J, Acuña-Alonzo V, et al A genome-wide association scan implicates DCHS2, RUNX2, GLI3, PAX1 and EDAR in human facial variation. Nat Commun. Nature Publishing Group; 2016;7:11616 10.1038/ncomms11616 27193062 PMC4874031

[39] Debat V, David P. Mapping phenotypes: canalization, plasticity and developmental stability. Trends Ecol Evol. 2001;16(10):555–61.

[40] Ercan I, Ozdemir ST, Etoz A, Sigirli D, Tubbs RS, Loukas M, et al Facial asymmetry in young healthy subjects evaluated by statistical shape analysis. J Anat. 2008;213(6):663–9. 10.1111/j.1469-7580.2008.01002.x 19094182 PMC2666135

[41] Farrera A, Villanueva M, Quinto-Sánchez M, González-José R. The relationship between facial shape asymmetry and attractiveness on mexican students. Am J Hum Biol. 2014;27(3):387–96. 10.1002/ajhb.22657 25400276

[42] Houle D. A simple model of the relationship between asymmetry and developmental stability. J Evol Biol. 2000;13(4):720–30.

[43] Palmer A. Fluctuating asymmetry analyses: A primer In: Markow T, editor. Developmental Instability: Its Origins and Evolutionary Implications. Kluwer, Dordrecht.; 1994 p. 335–64.

[44] Palmer A, Strobeck C. Fluctuating asymmetry analysis revisited In: Polak M., editor. Developmental instability Causes and consequences. Oxford, UK; 2003 p. 279–319. Oxford University Press

[45] Schmidt K, Cohn J. Human facial expressions as adaptations: Evolutionary questions in facial expression research. Am J Phys Anthropol. 2001;Suppl 33:3–24. 11786989 10.1002/ajpa.2001 PMC2238342

[46] Pellegrino A. Trends in International Migration in Latin America and the Caribbean. Int Soc Sci J. 2000;52(165):395–408.

[47] Trotsky L. The History of the Russian Revolution. Pr P, editor. Michigan: Pr, Pathfinder; 1932. 1040 p.

[48] Azevedo J, Inchaust G, Sanfelice V. Decomposing the Recent Inequality Decline in Latin America. World Bank Policy Research Working Paper No. 6715. 2013. Available at SSRN: https://ssrn.com/abstract=2365876

[49] CEPAL. Statistical Yearbook for Latin America and the Caribbean. 2012.

[50] Piot P, Greener R, Russell S. Squaring the circle: AIDS, poverty, and human development. PLoS Med. 2007;4(10):1571–5. 10.1371/journal.pmed.0040314 17958469 PMC2039763

[51] Ruiz-Linares A, Adhikari, Kaustubh Acuña-Alonzo V, Quinto-Sánchez, Mirsha Jaramillo C, Arias W, Fuentes M, Pizarro M, et al Admixture in Latin America: geographic structure, phenotipic diversity and self-perception of ancestry based on 7,342 individuals. PLoS Genet. 2014;10(9):e1004572 10.1371/journal.pgen.1004572 25254375 PMC4177621

[52] Quinto-Sánchez M, Adhikari K, Acuña-Alonzo V, Cintas C, Silva de Cerqueira CC, Ramallo V, et al Facial asymmetry and genetic ancestry in Latin American admixed populations. Am J Phys Anthropol. 2015;157(1):58–70. 10.1002/ajpa.22688 25582401

[53] Sigelman CK, Rider EA. Life-Span Human Development. Belmont, CA: Wadsworth Cengage Learning; 2009. 630 p.

[54] Purcell S, Neale B, Todd-Brown K, Thomas L, Ferreira MAR, Bender D, et al PLINK: a tool set for whole-genome association and population-based linkage analyses. Am J Hum Genet. 2007;81(3):559–75. 10.1086/519795 17701901 PMC1950838

[55] Yang J, Lee SH, Goddard ME, Visscher PM. GCTA: a tool for genome-wide complex trait analysis. Am J Hum Genet. 2011;88(1):76–82. 10.1016/j.ajhg.2010.11.011 21167468 PMC3014363

[56] Klingenberg CP, McIntyre GS. Geometric morphometrics of developmental instability: analyzing patterns of fluctuating asymmetry with Procrustes methods. Evolution (N Y). JSTOR; 1998;52(5):1363–75. 10.1111/j.1558-5646.1998.tb02018.x 28565401

[57] Singleton M. Patterns of cranial shape variation in the Papionini (Primates: Cercopithecinae). J Hum Evol. 2002;42(5):547–78. 10.1006/jhev.2001.0539 11969297

[58] Sokal RR, Rohlf FJ. Biometry: the principles and practice of statistics in biological research. San Francisco: W. H. Freeman; 1995. 880 p.

[59] Klingenberg CP, Barluenga M, Meyer A. Shape analysis of symmetric structures: quantifying variation among individuals and asymmetry. Evolution (N Y). 2002;56(10):1909–20. 10.1111/j.0014-3820.2002.tb00117.x 12449478

[60] Klingenberg CP. MorphoJ: an integrated software package for geometric morphometrics. Mol Ecol Resour. 2011;11(2):353–7. 10.1111/j.1755-0998.2010.02924.x 21429143

[61] Mardia K V, Bookstein FL, Moreton IJ. Statistical assessment of bilateral symmetry of shapes. Biometrika. 2000;87(2):285–300.

[62] Vyas S, Kumaranayake L. Constructing socio-economic status indices: How to use principal components analysis. Health Policy Plan. 2006;21(6):459–68. 10.1093/heapol/czl029 17030551

[63] Howe LD, Hargreaves JR, Huttly SRA. Issues in the construction of wealth indices for the measurement of socio-economic position in low-income countries. Emerg Themes Epidemiol. 2008;5(3):1–14. 18234082 10.1186/1742-7622-5-3 PMC2248177

[64] Palmer AR, Strobeck C. Fluctuating Asymmetry: Measurement, Analysis, Patterns. Annu Rev Ecol Syst. Annual Reviews 4139 El Camino Way, P.O. Box 10139, Palo Alto, CA 94303–0139, USA; 1986;17(1):391–421.

[65] Hallgrimsson B. Ontogenetic Patterning of Skeletal Fluctuating Asymmetry in Rhesus Macaques and Humans: Evolutionary and Developmental Implications. Int J Primatol. 1999;20(1):121–51.

[66] Farkas LG, Cheung G. Facial asymmetry in healthy North American Caucasians. An anthropometrical study. Angle Orthod. 1981;51(1):70–7. 10.1043/0003-3219(1981)051<0070:FAIHNA>2.0.CO;2 6939355

[67] Wilson JM, Manning JT. Fluctuating asymmetry and age in children: evolutionary implications for the control of developmental stability. J Hum Evol. 1996;30(1995):529–37.

[68] Ferrario VF, Sforza C, Miani A, Serrao G. A three-dimensional evaluation of human facial asymmetry. J Anat. 1995;186(Pt 1):103–10. 7649806 PMC1167276

[69] Smith WM. Hemispheric and facial asymmetry: gender differences. Laterality. 2000;5(3):251–8. 10.1080/713754376 15513145

[70] Koehler N, Simmons LW, Rhodes G, Peters M. The relationship between sexual dimorphism in human faces and fluctuating asymmetry. Proc Biol Sci. 2004;271 Suppl:S233–6. 10.1098/rsbl.2003.0146 15252993 PMC1810020

[71] Ferrario VF, Sforza C, Ciusa V, Dellavia C, Tartaglia GM. The effect of sex and age on facial asymmetry in healthy subjects: a cross-sectional study from adolescence to mid-adulthood. J Oral Maxillofac Surg. 2001;59(4):382–8. 10.1053/joms.2001.21872 11289167

[72] Claes P, Walters M, Shriver MD, Puts D, Gibson G, Clement J, et al Sexual dimorphism in multiple aspects of 3D facial symmetry and asymmetry defined by spatially dense geometric morphometrics. J Anat. 2012;221(2):97–114. 10.1111/j.1469-7580.2012.01528.x 22702244 PMC3406357

[73] Özener B. Does urban poverty increase body fluctuating asymmetry? Coll Antropol. 2011;35(4):1001–5. 22397230

[74] Rosero-Bixby L, Dow WH. Surprising SES Gradients in Mortality, Health, and Biomarkers in a Latin American Population of Adults. Journals Gerontol Ser B Psychol Sci Soc Sci. 2009;64B(1):105–17. 10.1093/geronb/gbn004 PMC2654981 19196695

[75] Marmot M, Wilkinson R. Social determinants of health. Oxford: Oxford University Press; 2005. 376 p.

[76] Aber JL, Bennett NG, Conley DC, Li J. The Effects of Poverty on Child Health and Development. Annu Rev Public Health. Annual Reviews 4139 El Camino Way, P.O. Box 10139, Palo Alto, CA 94303–0139, USA; 1997;18(1):463–83. 9143727 10.1146/annurev.publhealth.18.1.463

[77] Ramachandran S, Deshpande O, Roseman CC, Rosenberg NA, Feldman MW, Cavalli-Sforza LL. Support from the relationship of genetic and geographic distance in human populations for a serial founder effect originating in Africa. Proc Natl Acad Sci U S A. 2005;102(44):15942–7. 10.1073/pnas.0507611102 16243969 PMC1276087

[78] Vyas S, Kumaranayake L. Constructing socio-economic status indices: how to use principal components analysis. Health Policy Plan. 2006;21(6):459–68. 10.1093/heapol/czl029 17030551

[79] Lynch M, Walsh B. Genetics and Analysis of Quantitative Traits. Sunderland, MA: Sinauer Associates; 1998. 803 p.

