# Correction: Socioeconomic Status Is Not Related with Facial Fluctuating Asymmetry: Evidence from Latin-American Populations

**DOI:** 10.1371/journal.pone.0172418

**Published:** 2017-02-13

**Authors:** Mirsha Quinto-Sánchez, Celia Cintas, Caio Cesar Silva de Cerqueira, Virginia Ramallo, Victor Acuña-Alonzo, Kaustubh Adhikari, Lucía Castillo, Jorge Gomez-Valdés, Paola Everardo, Francisco De Avila, Tábita Hünemeier, Claudia Jaramillo, Williams Arias, Macarena Fuentes, Carla Gallo, Giovani Poletti, Lavinia Schuler-Faccini, Maria Cátira Bortolini, Samuel Canizales-Quinteros, Francisco Rothhammer, Gabriel Bedoya, Javier Rosique, Andrés Ruiz-Linares, Rolando González-José

The following information is missing from the Funding section: This work was partially supported by grant 207462 from the Fondo Cooperación Bilateral-Consejo Nacional de Ciencia y Tecnología (CONACyT)- Consejo Nacional de Investigaciones Científicas y Técnicas (CONICET).
