# Supplementary material for: Socioeconomic Status Is Not Related with Facial Fluctuating Asymmetry: Evidence from Latin-American Populations
Source: PLoS One. 2017 Jan 6;12(1):e0169287. doi: 10.1371/journal.pone.0169287 (PMC5218465; doi:10.1371/journal.pone.0169287)
Supplement: S1 Table — (DOCX) [file pone.0169287.s001.docx]

**S1 Table**

Sample characteristics regarding sample sizes, FFA mean values, standard deviation and variance by country and sex for heterozygosity corrected and uncorrected data.

|  | n | | |  | | | Mean | | | | | SD | | | | Variance | | |
| --- | --- | --- | --- | --- | --- | --- | --- | --- | --- | --- | --- | --- | --- | --- | --- | --- | --- | --- |
| Heterozigosity |  | | Uncorrected | | | Corrected | | | Uncorrected | | Corrected | | | Uncorrected | | | Corrected | |
| Sex | f | m | f | | m | f | | m | f | m | f | | m | f | m | | f | m |
| Brazil | 162 | 85 | 6.5366 | | 6.3847 | 0.2155 | | 0.0781 | 1.0465 | 0.9281 | 1.0448 | | 0.9418 | 1.0952 | 0.8613 | | 1.0915 | 0.8870 |
| Chile | 114 | 680 | 7.1768 | | 6.2085 | 0.6511 | | -0.3125 | 1.2648 | 1.0375 | 1.2366 | | 1.0394 | 1.5996 | 1.0764 | | 1.5291 | 1.0805 |
| Colombia | 314 | 233 | 6.0684 | | 6.0699 | -0.3131 | | -0.3112 | 0.9289 | 0.9716 | 0.9304 | | 0.9674 | 0.8629 | 0.9439 | | 0.8657 | 0.9360 |
| Mexico | 241 | 151 | 6.4315 | | 6.4700 | -0.1528 | | -0.0816 | 0.7840 | 0.9004 | 0.7861 | | 0.9145 | 0.6147 | 0.8108 | | 0.6179 | 0.8364 |
| Peru | 25 | 18 | 6.8323 | | 7.3836 | 0.2008 | | 0.7395 | 0.9570 | 0.8322 | 0.9891 | | 0.8754 | 0.9158 | 0.6926 | | 0.9783 | 0.7663 |
