# Supplementary material for: Socioeconomic Status Is Not Related with Facial Fluctuating Asymmetry: Evidence from Latin-American Populations
Source: PLoS One. 2017 Jan 6;12(1):e0169287. doi: 10.1371/journal.pone.0169287 (PMC5218465; doi:10.1371/journal.pone.0169287)
Supplement: S1 Text — (DOCX) [file pone.0169287.s003.docx]

**S1 Text**

Computation of a Wealth Index multivariate estimator**.**

Following the multivariate approach presented in references [1] and [2] (see below), we build a socio-economic model to characterize the Wealth Index (WI) in the sample departing from three variables available in the CANDELA survey: fixed monthly salary (FMS), frequency of domestic appliances by home, and education (schooling). The multivariate synthesis and ordination of such data was achieved using a principal component analysis (PCA), and retaining for further analyses just the PC axes exhibiting eigenvalues greater than one.. Four PC_WI_ axes achieve such condition, and are presented in Table SI-1), accounting 50.44% of the total variance. Figure SI-1 display the sorting of the original variables across the PC axes. The resulting PC scores were used as a WI into the context of the HLM approach (see text). The loading matrix depicting the contribution of the original variables on each PC is presented in Table SI-2.

Table SI-1. Principal Component Analysis of Wealth Index. Eigenvalues, percentage of explained variance and cumulative percentage of explained variance for each PC.

| No | Eigenvalue | % | Cum % |
| --- | --- | --- | --- |
| 1 | 4.2304 | 26.44 | 26.44 |
| 2 | 1.5963 | 9.977 | 36.417 |
| 3 | 1.2367 | 7.729 | 44.146 |
| 4 | 1.0082 | 6.301 | 50.447 |
| 5 | 0.9851 | 6.157 | 56.604 |
| 6 | 0.9412 | 5.883 | 62.487 |
| 7 | 0.8428 | 5.268 | 67.754 |
| 8 | 0.8009 | 5.005 | 72.76 |
| 9 | 0.7453 | 4.658 | 77.418 |
| 10 | 0.6765 | 4.228 | 81.646 |
| 11 | 0.6025 | 3.766 | 85.412 |
| 12 | 0.5686 | 3.554 | 88.966 |
| 13 | 0.4892 | 3.058 | 92.024 |
| 14 | 0.4755 | 2.972 | 94.996 |
| 15 | 0.4361 | 2.726 | 97.721 |
| 16 | 0.3646 | 2.279 | 100 |

Figure SI-1: Scatterplot of the first two PCs of WI. Arrows indicate the contribution of the original variable.


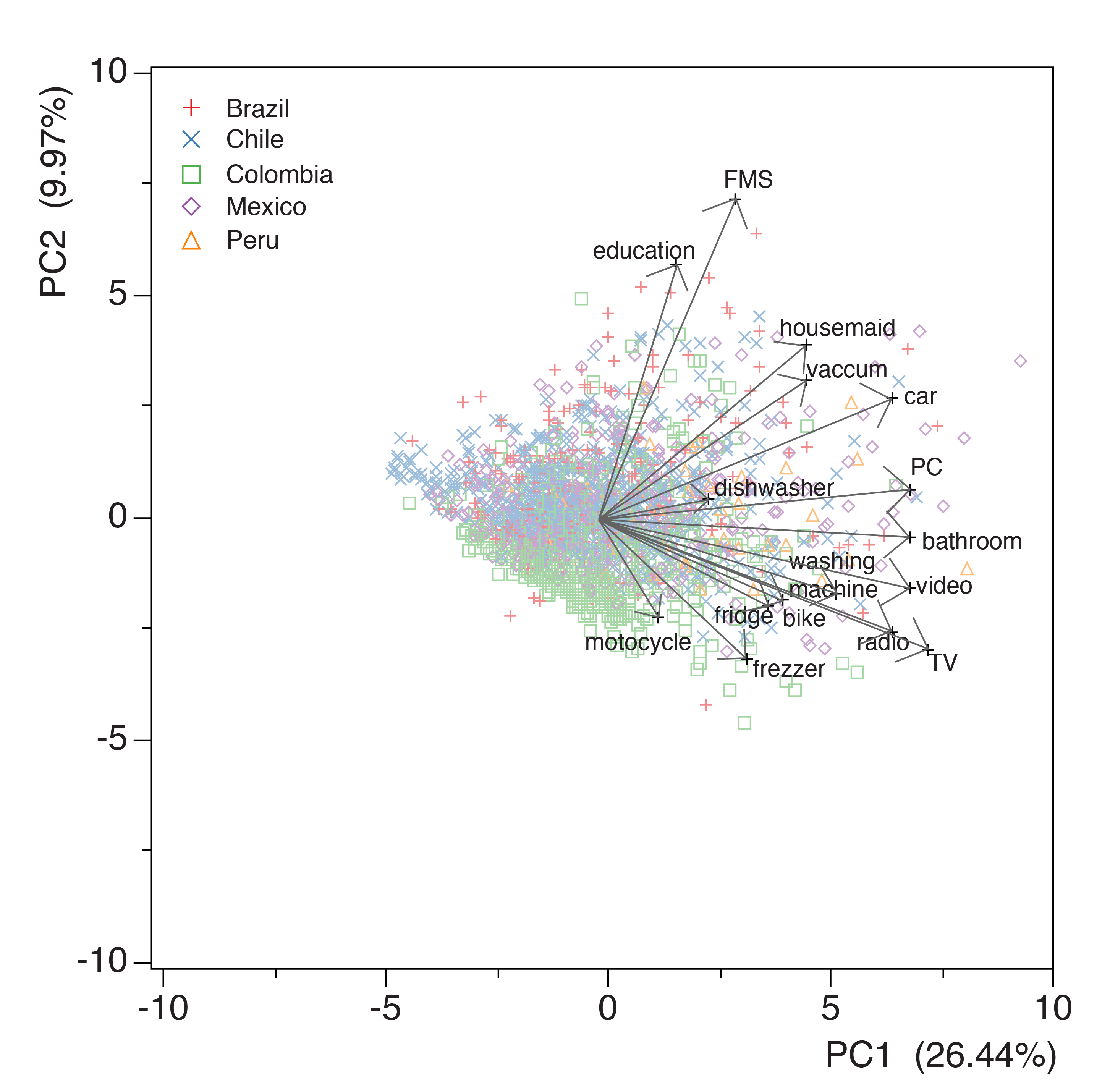


Table SI-2: Principal Component Analysis of Wealth Index. Loading matrix displaying the correlation between each original variable and the successive PCs.

|  | PC1 | PC2 | PC3 | PC4 |
| --- | --- | --- | --- | --- |
| TV | 0.740 | -0.291 | -0.120 | -0.065 |
| Radio | 0.657 | -0.250 | -0.050 | -0.129 |
| Bathroom | 0.698 | -0.039 | 0.258 | 0.137 |
| Car | 0.657 | 0.271 | -0.162 | -0.044 |
| Housemaid | 0.465 | 0.391 | 0.245 | 0.122 |
| Vaccum | 0.466 | 0.312 | -0.431 | 0.028 |
| Wash machine | 0.536 | -0.166 | -0.091 | 0.051 |
| Video | 0.699 | -0.154 | -0.197 | -0.154 |
| Fridge | 0.382 | -0.195 | 0.457 | 0.241 |
| Freezer | 0.330 | -0.312 | 0.299 | 0.273 |
| PC | 0.700 | 0.068 | 0.185 | -0.091 |
| Dishwaser | 0.247 | 0.045 | -0.232 | 0.707 |
| Motocycle | 0.134 | -0.219 | 0.381 | -0.446 |
| Bike | 0.415 | -0.183 | -0.318 | -0.242 |
| FMS | 0.308 | 0.722 | -0.068 | -0.146 |
| Education | 0.176 | 0.573 | 0.444 | -0.064 |

**SES variables in the univariate approach.**

SES variables have a country differences, that is, it have mean and some cases of variance differences by country. This is an expected result because differences in economy of the studied countries are evidenced by other economic indicators (e.g. Gini Index, what means a measure of statistical dispersion intended to represent the income distribution of a nation's residents commonly used measure of inequality). For this reason we nested all analysis by country and use the HLM model. We characterize the traditional approach to SES variables by pairwise correlation of corrected and uncorrected FFA scores for heterozygosity effect. In this results Table SI-3 emerge some significative correlations but with low variance explained. This table help us to explain the classic findings in the literature, were some effects of the SES variables are correlated with fluctuating asymmetry and presumable linked with development instability, but note that no one have high correlation or deep statistical signification. So, we are talking of a very small and specific part of the total variation in the sample. No there is no doubt that simple correlation of SES variables and FFA scores result in a univariate relationship but this effect disappears when we analyze data in multivariate way.

Table SI-3. Results of the pairwise univariate correlation of FFA scores with and without heterozygosity effect. Significative correlation is in blue, see column for p-values. All results shows lower correlations values.

| Sex | Pairwise | | Correlation | p |
| --- | --- | --- | --- | --- |
| Females | PC1_WI | Uncorrected FFA score | 0.043 | 0.213 |
|  |  | Corrected FFA score | 0.050 | 0.143 |
|  | PC2_WI | Uncorrected FFA score | 0.143 | <.0001 |
|  |  | Corrected FFA score | 0.140 | <.0001 |
|  | PC3_WI | Uncorrected FFA score | -0.106 | 0.002 |
|  |  | Corrected FFA score | -0.053 | 0.121 |
|  | PC4_WI | Uncorrected FFA score | -0.062 | 0.069 |
|  |  | Corrected FFA score | -0.064 | 0.062 |
| Males | PC1_WI | Uncorrected FFA score | 0.077 | 0.009 |
|  |  | Corrected FFA score | 0.089 | 0.002 |
|  | PC2_WI | Uncorrected FFA score | 0.038 | 0.199 |
|  |  | Corrected FFA score | 0.028 | 0.347 |
|  | PC3_WI | Uncorrected FFA score | 0.008 | 0.784 |
|  |  | Corrected FFA score | 0.046 | 0.119 |
|  | PC4_WI | Uncorrected FFA score | 0.018 | 0.547 |
|  |  | Corrected FFA score | 0.014 | 0.626 |
|  |  |  |  |  |

Differences of FFA scores by country.

For explore the tendency of fluctuating asymmetry by country we implement a one way ANOVA and pos hoc Tukey-Kramer test for pairwise comparisons. Results show that data are population structured (p <0.0001, see Table SI-4), pos hoc test let us know which iterations represents a statistical significative differences.

Table SI-4. Pos hoc Tukey-Kramer test for structuration by country in the sample. Blue cell indicate statistical differences at α=0.01 level.

|  |  | Uncorrected FFA score | | |  | Corrected FFA score | | |
| --- | --- | --- | --- | --- | --- | --- | --- | --- |
| Sex | Iteration | Difference | se | p |  | Difference | se | p |
| Females | CHI-COL | 1.108 | 0.106 | <.0001 |  | 0.964 | 0.105 | <.0001 |
|  | PER-COL | 0.764 | 0.201 | 0.002 |  | 0.804 | 0.110 | <.0001 |
|  | CHI-MEX | 0.745 | 0.110 | <.0001 |  | 0.529 | 0.093 | <.0001 |
|  | CHI-BRA | 0.640 | 0.118 | <.0001 |  | 0.514 | 0.200 | 0.078 |
|  | BRA-COL | 0.468 | 0.094 | <.0001 |  | 0.450 | 0.213 | 0.215 |
|  | PER-MEX | 0.401 | 0.203 | 0.281 |  | 0.436 | 0.118 | 0.002 |
|  | MEX-COL | 0.363 | 0.083 | 0.000 |  | 0.368 | 0.098 | 0.002 |
|  | CHI-PER | 0.344 | 0.214 | 0.490 |  | 0.354 | 0.203 | 0.407 |
|  | PER-BRA | 0.296 | 0.208 | 0.613 |  | 0.160 | 0.083 | 0.296 |
|  | BRA-MEX | 0.105 | 0.098 | 0.823 |  | 0.015 | 0.207 | 1.000 |
| Males | CHI-COL | 1.314 | 0.244 | <.0001 |  | 1.052 | 0.239 | 0.000 |
|  | PER-COL | 1.175 | 0.238 | <.0001 |  | 1.051 | 0.245 | 0.000 |
|  | CHI-MEX | 0.999 | 0.259 | 0.001 |  | 0.821 | 0.250 | 0.009 |
|  | CHI-BRA | 0.914 | 0.249 | 0.002 |  | 0.661 | 0.260 | 0.081 |
|  | BRA-COL | 0.400 | 0.104 | 0.001 |  | 0.391 | 0.115 | 0.006 |
|  | PER-MEX | 0.315 | 0.126 | 0.093 |  | 0.389 | 0.127 | 0.019 |
|  | MEX-COL | 0.261 | 0.090 | 0.030 |  | 0.231 | 0.090 | 0.078 |
|  | CHI-PER | 0.176 | 0.115 | 0.539 |  | 0.230 | 0.105 | 0.182 |
|  | PER-BRA | 0.139 | 0.076 | 0.356 |  | 0.160 | 0.136 | 0.765 |
|  | BRA-MEX | 0.085 | 0.135 | 0.970 |  | 0.001 | 0.076 | 1.000 |

**References**

1. Vyas, S. & Kumaranayake, L. 2006 Constructing socio-economic status indices: how to use principal components analysis. *Health Policy Plan.* **21**, 459–68. (doi:10.1093/heapol/czl029)

2. Howe, L. D., Hargreaves, J. R. & Huttly, S. R. A. 2008 Issues in the construction of wealth indices for the measurement of socio-economic position in low-income countries. *Emerg. Themes Epidemiol.* **5**, 1–14. (doi:10.1186/1742-7622-5-3)
